# Le pronostic statural après une pseudo-puberté précoce par déficit en 11ß hydroxylase chez une fille de 7 ans: à propos d'un cas avec revue de la literature

**DOI:** 10.11604/pamj.2015.20.162.5905

**Published:** 2015-02-23

**Authors:** Hanane Latrech, Ahmed Gaouzi

**Affiliations:** 1 Department of Endocrinology, Mohammed VI hospital, Medical school, University Mohamed First, Oujda, Morocco; 2 Department of Endocrinology Paediatrics, Medical school, Mohammed V Souissi University, Ibn Sina Hospital, Rabat, Morocco

**Keywords:** 11ß hydroxylase, puberté précoce, analogues de LH-RH, 11ß hydroxylase, early puberty, LH RH analogs

## Abstract

L'hyperplasie congénitale des surrénales par déficit en 11ß hydroxylase se voit dans 5 à 10% des cas et est diagnostiquée, habituellement, devant des signes de virilisation d'un nouveau né ou d'un fœtus 46 XX et plus tard devant une HTA avec une hypokaliémie. Le pronostic de taille chez ces patients est très souvent compromis. Nous rapportons le cas d'un déficit en 11ß hydroxylase chez une enfant âgée de 7 ans révélé par une pseudo- puberté précoce traitée par hydrocortisone. La survenue d'une puberté précoce centrale complique encore plus la situation par la mise en jeu du pronostic de taille finale et nécessitant une prise en charge thérapeutique particulière.

## Introduction

L'hyperplasie congénitale des surrénales (HCS) est une maladie endocrinienne génétique à transmission autosomique récessive résultant d'un déficit d'une des enzymes de la stéroïdogénèse responsable de la synthèse du cortisol. Le déficit en 11ß hydroxylase est le plus fréquent après celui en 21 hydroxylase et représente 5 à 8% des HCS [1]. Le pronostic de taille finale chez ces patients peut être, parfois, un enjeu difficile à assurer et doit faire l'objet d'une attention particulière par le clinicien pouvant faire appel à des compléments thérapeutiques indispensables au traitement classique par glucocorticoïdes. Nous rapportons le cas d'un déficit en 11ß hydroxylase diagnostiqué à l’âge de 7 ans et illustrant les difficultés d’évaluation et de prise en charge lorsque le pronostic de taille finale est compromis.

## Patient et observation

**Figure 1:**
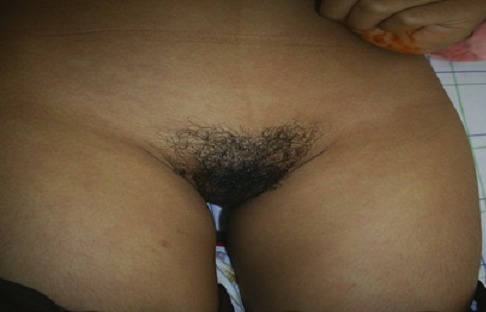
Pilosité pubienne

**Figure 2:**
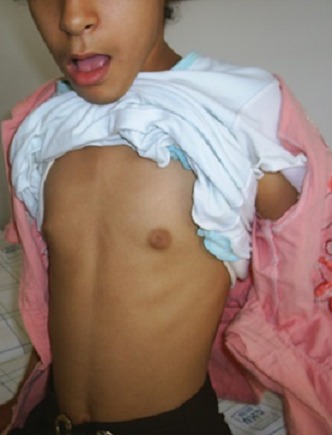
Développement mammaire lors de la puberté précoce centrale

Il s'agit d'une patiente âgée de 7ans, qui a comme antécédent une consanguinité 1ère degré, consultant pour l'apparition d'une pilosité pubienne et axillaire installée deux ans auparavant. L'examen clinique trouve un développement mammaire (S1-S2), une pilosité pubienne (P3-4) (Figure 1) et axillaire (P1-2), un hirsutisme localisé surtout au niveau du visage et du dos, une avance staturale (+2 DS) sans hypertrophie clitoridienne ni de métrorragies. La tension artérielle est de 130/ 80 mmHg. L’âge osseux évalué selon Greulish et pyle est de 11 ans. Un bilan hormonal fait a montré un taux de testostéronémie à 1.74 ng/ml, un taux de FSH à 4.3 mUI/ml (VN: 0.3-11.1), un taux de LH inférieure à 0.4 mUI/ml et un taux d’œstradiol inférieur à 20 pg/ml. Le dosage de la 17 hydroprogestérone est de 2.4 ng/ml avec un taux de désoxycorticostérone très élevé 3300 ng/ml et une 11 désoxycortisol élevée. L’échographie abdomino- pelvienne montre une hyperplasie des deux surrénales et un utérus impubère. La patiente a été donc mise sous hydrocortisone à la dose de 10 mg/jour avec normalisation du bilan hormonal. A l’âge de 8 ans et malgré une bonne observance thérapeutique, elle a présenté une évolutivité des signes pubertaires (Figure 2) avec à l'examen clinique des seins stade 2-3 de Tanner, une pilosité pubienne (P3-4), une pilosité axillaire (2-3), une avance staturale (+2+3) sans hypertrophie clitoridienne ni de métrorragies. Les taux de gonadotrophines a augmenté avec un taux de la FSH à 3,07 mUI/ml, un taux de LH à 4.2 mUI/ml et celui de l’œstradiol à 39.6 pg/ml (VN: 6-27). L’âge osseux est de 11 ans. L’échographie pelvienne montre un utérus mesurant 34×24×17 mm avec des ovaires droit et gauche mesurant respectivement 21×12 mm et 22×14 mm. Un test de stimulation à la LH-RH a montré un pic de LH à 33.6 mUI/ml et au pic de FSH à 16.6 mUI/ml concluant donc à une puberté précoce centrale vraie par levée d'inhibition. La taille cible est de 159 cm avec une taille prédite (en retranchant 5cm de la taille cible par année d'avance osseuse) est de 144 cm.

## Discussion

L'hyperplasie congénitale des surrénales est due dans 5 à 8% des cas à un déficit en 11ß hydroxylase. Son incidence est d'environ 1/200 000 dans la population générale dont un grand nombre a été rapporté dans la population juive d'origine marocaine [1]. Environ 160 cas ont été rapportés dans la littérature mais avec seulement 60 cas qui ont été confirmés par biologie moléculaire [2]. Ce déficit enzymatique est responsable d'un défaut de synthèse du cortisol et de l'aldostérone avec une accumulation des métabolites en amont, soit le composé S et le désoxycorticostérone (DOC) produits dans la fasciculée-réticulée suite à l'hypersécrétion de l'ACTH, et un excès de synthèse des androgènes surrénaliens par la seule voie métabolique possible [1]. De ce fait, la clinique du déficit en 11ß hydroxylase diffère de celle en 21 hydroxylase par l'hypersécrétion du DOC et ses métabolites connus par leur action minéralocorticoïde pouvant générer une hypertension artérielle qui reste non corrélée aux taux du DOC mais pouvant être responsable de complications graves à type de rétinite, de néphropathie hypertensive d'hypertrophie ventriculaire gauche ou d'accidents vasculaires cérébraux [1, 3, 4]. Chez la fille, le diagnostic se fait à la naissance devant une virilisation des organes génitaux externes sans perte de sel. Cette forme ressemble à la forme virilisante pure du bloc en 21-hydroxylase sans qu'elle soit dépistée lors du dépistage néonatal. Chez le garçon, le diagnostic est souvent tardif et sera fait devant un tableau de pseudo-puberté précoce survenant le plus souvent avant l’âge de 3 ans [1, 4]. Malgré l'absence de déficit en minéralocorticoïdes, certains syndromes de perte de sel ont été décrits pouvant survenir surtout au décours du traitement par l'hydrocortisone [1, 4]. En effet, ce traitement inhibe la sécrétion des précurseurs à savoir le DOC qui avait auparavant bloqué le système rénine angiotensine sans que la synthèse de l'aldostéronene soitdirectement affectée. Chez notre patiente, le diagnostic a été fait devant un tableau de pseudo-puberté précoce associant une pilosité pubienne et axillaire et un hirsutisme avec une HTA limite, une accélération de la vitesse de croissance et une avance d’âge osseux. L'accélération de la maturation osseuse aboutit à une fusion précoce des cartilages de conjugaison et donc à un risque de petite taille finale. Sur le plan biologique, le diagnostic du déficit en 11ß hydroxylase est évoqué par l'augmentation des taux sériques de la 11-désoxycortisol et du DOC, de base et après synacthène, associées à un taux de rénine plasmatique bas [1, 4].

Parfois une hypokaliémie, associée à une myopathie ou une rabdomyolyse, est retrouvée mais de manière moins fréquente que dans les autres causes d'HTA à rénine basse [1, 5]. Devant des taux un peu élevés de 17OH progestérone, comme était le cas chez notre patiente, le rapport delta 4 androsténédione / 17 OH progestérone est très utile en attendant le résultat du composé S et du DOC. En effet, ce rapport est toujours supérieur à 1 dans les déficits en 11ß hydroxylase alors qu'il est effondré dans les déficits en 21 hydroxylase (<0.5). Cette augmentation préférentielle de la delta 4 s'explique par le fait que la 11ß hydroxylase inactive la delta4 en 11ß androsténedione [1]. L’étude moléculaire signe le diagnostic de certitude en mettant en évidence la présence de mutation au niveau du gène CYP11B1 localisé sur le chromosome 8 et qui est exprimé uniquement dans la zone fasciculée et est modulé par l'ACTH. Il est rapporté une bonne corrélation entre le génotype et le phénotype. A ce jour, plus de 50 mutations différentes ont été décrites [1, 4]. La mutation R448H est la plus fréquente notamment chez les immigrants juifs d'origine marocaine [6]. Récemment, de nouvelles mutations du gène CYP11B1 ont été décrites [7]. Le traitement consiste seulement à la prise d'hydrocortisone, qui n'est pas une urgence chez le nouveau né du fait de l'absence de syndrome de perte de sel et l'apparition tardive de l'HTA, à la dose de 10 à 20 mg/m^2^/jour en trois prises à adapter sur des éléments clinico-biologique (la croissance staturale et pondérale, les signes d'hyperandrogénies, la pression artérielle, le composé S, le DOC, les androgènes et la rénine) [1, 4]. L'instauration de ce traitement substitutif peut entrainer, chez les enfants de 7 à 8 ans et ayant une avance d’âge osseux dépendante de l’œstrogénisation précoce de la plaque de croissance, une puberté précoce centrale qui réduit encore plus le temps de croissance de ces patients dont le pronostic de taille finale est connu déjà compromis et réduit d'approximativement 10 cm par rapport à leur taille cible avec présence de corrélation directe entre le dégrée de l’équilibre hormonal et le dégrée de la perte staturale [4, 8, 9]. En effet, et comme est le cas aussi chez notre patiente, l'imprégnation plus au moins longue par les androgènes conduit à une maturation somatique et osseuse qui va modifier le contrôle de l'axe gonadotrope avec installation d'une puberté précoce vraie centrale après la mise en route d'un traitement par glucocorticoïdes qui va freiner la production d'androgènes surrénaliens et donc lèvera leur inhibition sur l'axe gonadotrope. A ces mécanismes, s'y ajoute le traitement glucocorticoïde, comme facteur pouvant participer à la réduction de taille finale en diminuant la sécrétion endogène de la GH et la bioactivité de l'IGF1 [8, 9]. De ce fait, le pronostic de taille chez les HCS doit toujours faire l'objet d'une grande vigilance en commençant par l’évaluation de l’âge osseux du poignet qui, même si avancé, ne prédit pas malheureusement la maturation de la plaque de croissance du fémur inférieur et des vertèbres qui représente les principaux éléments de l'accroissement de la stature à la fin de l'enfance et l'adolescence. On se contente donc d'approximations pour apprécier la taille prédite. Le calcul de la taille cible, à partir de la taille des deux parents, est aussi un élément important pour pronostiquer la taille adulte [10]. Le pronostic de taille était en plus aggravé, chez notre patiente, par la petite taille parentale à laquelle il faut prêter la plus grande attention. Cette activation hypothalamo- hypophysaire, marquée par l'apparition ou une évolutivité des signes pubertaires cliniques et échographiques, doit être systématiquement confirmée par un test au LH-RH. Et au vue de ce test, une démarche polythérapeutique adaptée et à dose efficace doit être entamée. Mais le clinicien doit s'assurer auparavant de la normalisation des chiffres tentionnels et de la qualité du freinage glucocorticoïde de l'ACTH qui n'est pas toujours facile surtout si le patient est plus compliant les jours où les dosages sont faits. Il est aussi indispensable de répartir une dose suffisante de glucocorticoïde en 3 à 4 prises à fin d’éviter, lors d'une augmentation maladroite des doses répartis en deux prises, les effets secondaires de la corticothérapie dans les moments trop traités et la poursuite de la maturation osseuse dans les moments où l'effet des glucocorticoïdes ne s'exerce plus. En plus du pronostic statural mis en jeu, le freinage de cette puberté précoce est capital du fait des modifications psychologiques qu'elle risque d'entrainer chez un enfant qui est encore immature [1, 4, 8, 9]. Ce blocage fera appel à des agonistes de la gonadolibérine: Triptoréline (décapeptyl 3mg) ou Leuproréline (Enantone 3.75 mg) toutes les 4 semaines en intramusculaire (à la dose d´une ½ ampoule chez un enfant de moins de 20 kg et une ampoule complète au-delà.) avec une bonne efficacité. Ce traitement est généralement maintenu jusqu´à l’âge osseux de 12 ans et 6 mois chez la fille et 13 ans chez le garçon. A l'arrêt de ce traitement, on s'attend à voir habituellement un 2ème pic statural qui, dans la plupart des cas, est malheureusement de très faible amplitude contribuant ainsi à diminuer la taille définitive. De ce fait, certains auteurs ont proposé l'adjonction d'un traitement par l'hormone de croissance (0.33 mg/kg/semaine) juste avant le versant ascendant du pic statural pubertaire. Cette possibilité thérapeutique, utilisée hors AMM, doit être instaurée au bon moment alors qu'il reste encore un potentiel de croissance satisfaisant [4, 8, 9, 11, 12]. Plusieurs auteurs ont rapporté un bénéfice réel de cette option thérapeutique, sur la taille finale, comme un complément indispensable du traitement par la LH-RH dans l'HCS par bloc en 11ß hydroxylase [11, 13]. Chez notre patiente, il a été proposé d´instaurer un traitement bloquant la puberté centrale mais cette option thérapeutique n´a pas été accepté par les parents.

## Conclusion

Le déficit en 11ß hydroxylase est une entité pathologique à transmission autosomique récessive encore méconnue. La survenue d'une puberté précoce chez ces patients complique encore plus leurs situations et implique une thérapeutique particulière associant des agonistes de LH-RH et l´hormone de croissance comme complément qui pourrai s´ajouter au traitement classique de l'HCS afin d'améliorer le pronostic de taille finale et d’éviter les complications psychologiques inhérentes à la survenue précoce de la puberté.
